# Trajectories of Cancer Antigen 125 (CA125) Within 3 and 6 Months After the Initiation of Chemotherapy Treatment for Advanced Ovarian Cancer and Clinical Outcomes: A Secondary Analysis of Data from a Phase III Clinical Trial

**DOI:** 10.3390/curroncol32070390

**Published:** 2025-07-07

**Authors:** Chang Yin, Josee-Lyne Ethier, Mark S. Carey, Dongsheng Tu, Xueying Zheng

**Affiliations:** 1 Department of Biostatistics, School of Public Health, Fudan University, and the Key Laboratory of Public Health Safety of Ministry of Education, Shanghai 200032, China; ashley__yin@163.com; 2 Odette Cancer Centre, Sunnybrook Health Sciences Centre, Toronto, ON M4N 3M5, Canada; joseelyne.ethier@sunnybrook.ca; 3 Division of Gynecologic Oncology, Department of Obstetrics and Gynecology, University of British Columbia, Vancouver, BC V6H 3N1, Canada; mark.carey@vch.ca; 4 Canadian Cancer Trials Group, Queen’s University, Kingston, ON K7L 3N6, Canada; 5 Shanghai Institute of Infectious Disease and Biosecurity, Shanghai 200032, China

**Keywords:** CA125, prognosis, progression-free survival, overall survival, trajectories

## Abstract

Background: A single measurement or a summary of a limited number of measurements of CA125 was considered in the prediction of clinical outcomes for patients with ovarian cancer. We aimed to identify the classes of patients with advanced ovarian cancer based on their CA125 trajectory and to investigate the heterogeneity of clinical outcomes among the patients in the different classes. Methods: CA125 trajectory classes were identified by latent-class mixed models based on values collected within 3 and 6 months post-treatment for 819 women with advanced ovarian cancer enrolled in a randomized trial. Results: Based on their CA125 values during the first 6 months of treatment, the patients with low CA125 levels at baseline that remained low during treatment had the best clinical outcome (a median survival of 83 months and a progression-free survival of 34 months). In contrast, the patients with high CA125 values at baseline with a modest decrease during treatment had the highest risk of death and progression (hazard ratio [95% confidence interval]: 4.83 [3.56, 6.54] for overall survival and 5.15 [3.87, 6.87] for progression-free survival). Conclusions: Longitudinal trajectories of CA125 may provide more direct information for the prognoses of patients with advanced ovarian cancer undergoing chemotherapy treatment.

## Simple Summary

CA-125 (cancer antigen 125) is a cell surface protein that can be measured in the blood. Levels of CA-125 are often increased in ovarian cancers that express this protein. Previous studies suggested that a single measurement or a summary of a limited number of measurements of the levels of CA125 may be used to forecast the results of treatments for patients with ovarian cancer. In this study, we found that the patterns of change in the levels of CA125 within three or six months after the start of chemotherapy provide more direct information on the results of chemotherapy treatment. Specifically, our study demonstrated a decreased risk of progression and death for patients whose CA125 levels were low at the start of the treatment and remained low during the treatment and an increased risk when patients’ CA125 levels remained elevated. This information may be useful for patient monitoring.

## 1. Introduction

Ovarian cancer is the third most common and the most lethal onco-gynecological disease in the world [1]. The 5-year survival rate for early-stage and late-stage cancer is approximately 90% and 20–40%, respectively [2,3]. Most women are diagnosed at the advanced stage. In patients with advanced ovarian carcinomas, the standard first-line treatment typically includes debulking surgery and platinum-based (carboplatin/paclitaxel) chemotherapy. Although treatment significantly improves survival, the improvement is modest, and most women will experience cancer recurrence and eventually die of their disease. The prediction of clinical outcomes for women undergoing front-line treatment is important to guide treatment and aid in clinical decision making.

CA125 is an important biomarker in ovarian cancer. Many studies have investigated its value in the prediction of clinical outcomes for patients with ovarian cancer. For example, a nomogram based on the baseline CA125 level and other clinical factors was developed to predict the 3-year recurrence risk in patients with epithelial ovarian cancer who achieved clinical complete remission after cytoreductive surgery and chemotherapy [4]. The CA125 nadir, defined as the first measurement of CA125 within one month after first-line treatment, was found to be related to progression-free survival but not to overall survival in patients who achieved complete remission [5]. Recently, a strong association was found between the KELIM (modeled CA125 elimination rate constant K), which is derived from the CA125 longitudinal kinetics during the first 100 days of neo-adjuvant or adjuvant chemotherapy, and survival [6]. This result was also confirmed recently in a meta-analysis of clinical trials conducted in the first-line setting before the poly(ADP-ribose) polymerase inhibitor era [7]. Various approaches summarizing the early trajectory of CA-125 at 3 or 6 months after treatment initiation, which include the observed and estimated measures obtained by a linear mixed model (LMM), were also compared recently in an individual patient data meta-analysis [8].

The majority of previous studies have only considered a single CA125 measurement or a limited number of measurements taken at well-defined times and ignored the developmental trajectories of CA125. We hypothesize that the dynamic changing patterns during the treatment period can provide more direct and visible information on the relationships of CA125 with the clinical outcomes of ovarian cancer patients. Based on this hypothesis, this study aims to identify the trajectory class of CA125 within three and six months after the start of chemotherapy treatment, which are at the midway point, and at the end of treatment respectively and to evaluate the prognostic value of the longitudinal trajectory classes identified.

## 2. Materials and Methods

### 2.1. Study Design

The data for this study were collected in a Canadian Cancer Trials Group OV.16 trial (clinical trial registration number: NCT00028743) involving 819 women with stage-IIB or higher ovarian cancer. Between 31 August 2001 and 29 June 2005, the patients in this study were randomly assigned to two different chemotherapy regimens: one included four cycles of cisplatin 50 mg/m^2^ on day 1 and topotecan 0.75 mg/m^2^ on days 1–5 followed by four cycles of paclitaxel 175 mg/m^2^ over 3 h on day 1 and carboplatin (area under the curve = 5) on day 1 (arm 1); the other included eight cycles of paclitaxel 175 mg/m^2^ over 3 h on day 1 followed by carboplatin (area under the curve = 5) on day 1 (arm 2). Both of these regimens were administered as first-line chemotherapy following primary surgery or before interval debulking [9]. Each participating institution obtained the required ethical or institutional review board approval to enroll patients in this study. At baseline, the median age of the patients was 57 years (range = 28–78); 81% had debulking surgery, and the residual disease was less than 1 cm among 55% of them; the disease stage was III for 66% of the patients; and 388 (47.4%) patients had measurable disease. Progression-free survival (PFS) was the primary endpoint of the trial, which was defined as the time from randomization to the earliest time when the disease’s progression was observed or death without progression was documented. The overall survival (OS), defined as the time from the date of randomization to death from any cause, was included as a secondary endpoint. Serum CA125 was obtained on day 1 of each cycle, every 3 months for the first 3 years after the end of treatment, then every 6 months for a further 2 years, and then annually. More frequent investigations were permitted if medically indicated. Because no significant difference was found in the PFS or the OS between the two treatment arms, the patients in the two treatment arms were combined in this analysis.

### 2.2. Statistical Analysis

A latent-class mixed model (LCMM), as implemented in the “lcmm” package (version 1.9.3) of R (version 4.1.0), was applied to identify the different developmental trajectories of the CA125 values, which, due to non-normality, fitted the logarithms of the transformed CA125 measurements using a linear mixed model for the estimation of latent classes [10,11]. To determine the optimal number and shape of the trajectory classes, we set the measurements of CA125 as linear, quadratic, or cubic functions of time (the months between each measurement and the baseline) in the models and went through 1–6 latent classes [12]. The selection criteria included the Bayesian information criterion, the acceptable proportion of the population, and the posterior probability.

The baseline characteristics of patients across different CA125 trajectory classes were described as the median [range] and compared using Kruskal–Wallis tests for the continuous variables, and they were described as a number (%) and compared using chi-square tests for the categorical variables. The overall survival (OS) and the progression-free survival (PFS) of patients in different CA125 trajectory classes were described by Kaplan–Meier curves and compared by multivariate Cox proportional hazard models, adjusting for following clinically important baseline factors: age (≤65 vs. >65); the extent of pre-randomization surgery (no macroscopic residual or a macroscopic residual < 1 cm vs. a macroscopic residual ≥ 1 cm vs. no debulking); the FIGO stage (II vs. III or IV); the grade (well or moderate vs. poor, undifferentiated, or unknown); histology (serous adenocarcinoma vs. others); and the ECOG performance status (0 vs. 1). The prognostic value of the CA125 trajectory class was evaluated by the C-index, which estimates the probability that the predicted results are consistent with the actual observed value. For all comparisons, two-sided *p* < 0.05 was considered as the threshold for statistical significance. All the statistical analyses were performed with R software (version 4.1.0).

## 3. Results

### 3.1. Classification Based on CA125 Values Within 3 Months After Treatment

**Figure 1:**
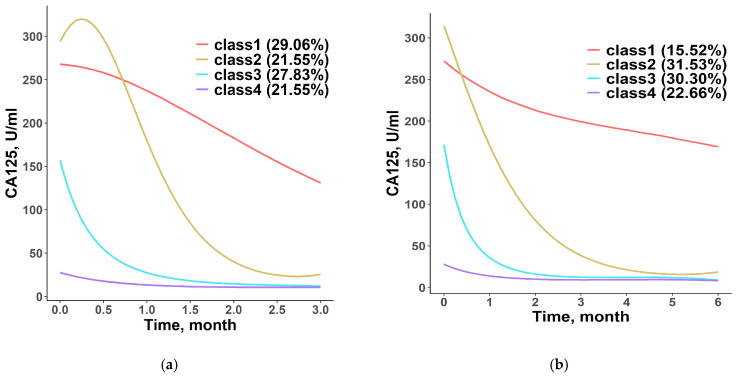
CA125 trajectory in identified patient classes based on values collected within 3 months (**a**) and 6 months (**b**) post-treatment.

Four CA125 trajectory classes were identified by an optimal model with cubic curves from 812 patients, with 3334 CA125 measurements obtained within 3 months post-randomization (Figure 1a): 236 (29.1%) patients in Class 1 had a high CA125 value at baseline but a very moderate decrease afterwards, while 175 (21.6%) patients in Class 2 had a high CA125 value at baseline and a sharp decrease after a small increase at around 1 week after randomization. Class 3 includes 226 (27.8%) patients with moderate CA125 value at baseline, which decreased quickly to a very low level after around 1 month after randomization; the CA125 value was very low at baseline for 175 (21.6%) patients in Class 4 and remained stable.

The baseline characteristics of the patients in these four classes are summarized in Table 1. The patients in Class 1 were the oldest (median 58.9 years with a range from 30.9 to 78.3 years).

**Figure 2:**
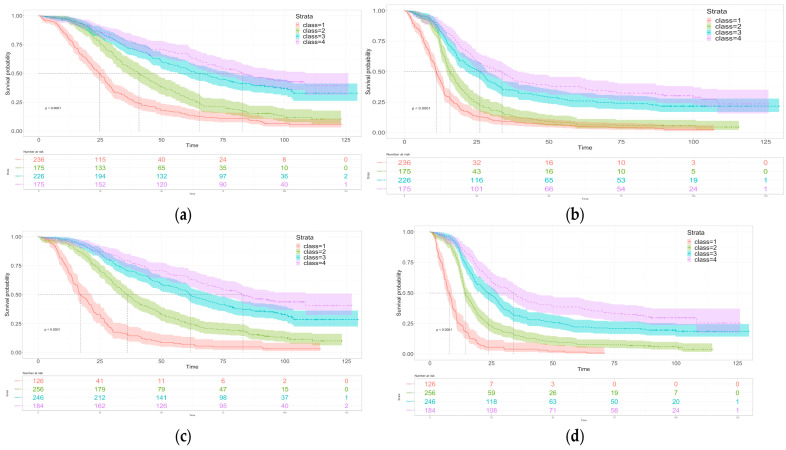
Kaplan–Meier Curves by 3-month CA125 trajectory class for OS (**a**) and PFS (**b**) and by 6-month CA125 trajectory class for OS (**c**) and PFS (**d**).

Kaplan–Meier curves of the OS and the PFS for the trajectory groups are shown in Figure 2a,b. The median OS values were 24.7, 40.8, 65.4, and 83.0 months, respectively, for the patients in Class 1 to Class 4, with corresponding median PFS values of 11.0, 15.7, 26.1, and 33.8 months.

After adjusting for baseline characteristics (age, the extent of pre-randomization surgery, stage, grade, histology, and ECOG performance), the patients in Class 1 and Class 2 had a significantly higher risk of death (hazard ratio 3.13 (2.37, 4.13) with 95% CI from 2.37 to 4.13 and *p* < 0.001 in Class 1 and 1.86 with 95% confidence interval from 1.40 to 2.46 and *p* < 0.001 in Class 2) compared to those in Class 4. The risk of death was not significantly different between the patients in Class 3 and Class 4 (hazard ratio 1.07 with 95% confidence interval from 0.82 to 1.41 and *p* = 0.614) (Table 2). Similar conclusions can be drawn for the associations between the CA125 trajectory class and the PFS (Table 2).

To assess the use of the CA125 trajectories observed at 3 months for the prediction of clinical outcomes beyond 3 months, a landmark analysis was performed by removing the events observed before 3 months following the randomization. Similar results were observed for the association between the CA125 trajectory class and the clinical outcomes after the landmark times, compared with the analyses of clinical outcomes from randomization (Figure S1 and Table S1). The C-index for the measure of the prognostic value of the CA125 trajectory class was 0.679 for the OS and 0.670 for the PFS.

### 3.2. Classification Based on CA125 Values Within 6 Months After Treatment

Four classes were also identified by an optimal model with cubic curves from 812 patients with 5996 CA125 measurements made within 6 months after treatment (Figure 1b). In Class 1, 126 (15.5%) patients started from about 300 U/mL and decreased very slowly over 6 months. The CA125 value of 256 (31.5%) patients in Class 2 had high baseline CA125 values comparable to those in Class 1 but decreased sharply in three months and then maintained a stable level at around 25 U/mL. For 246 (30.3%) patients in Class 3, values started at approximately 150 U/mL and decreased to the same level as the patients in Classes 1 and 2 within about 2 months. The CA125 value of 184 (22.7%) patients in Class 4 was within the upper normal limit during the 6 months.

The baseline characteristics of the patients in these four classes, presented in Table 3, show that those in Class 2 were the oldest (median 58.9 years with a range from 35.5 to 75.9 years), while the patients in Class 4 were the youngest (median 55.6 years with a range from 30.6 to 76.0 years). Most of the patients in Class 1, but least in Class 4, had an ECOG performance status of one (80.9% and 48.4%, respectively) and stage-IV disease (47.6% and 4.9%, respectively). Most of the patients in Class 3 and least in Class 4 had serous adenocarcinoma (76.0% and 55.4%, respectively).

From the Kaplan–Meier curves presented in Figure 2c,d, the median OS values for the patients in Classes 1 to 4 were 17.3, 36.1, 61.7, and 83.0 months, respectively, and the corresponding the median PFS values were 7.9, 14.5, 23.7, and 34.0 months. The patients in Classes 1 and 2 had a significantly higher risk of death than those in Class 4 (HR 4.83 [3.56, 6.54] and *p* < 0.001 between Classes 1 and 4; 1.98 with 95% CI and *p*-value 1.98 [1.52 to 2.60] and *p* < 0.001 between Classes 2 and 4), but the OS was not significant between Classes 3 and 4 (HR 1.18 with 95% CI 0.91 to 1.54 and *p*-value 0.219) (Table 2). Similar associations between the CA125 trajectory class and the PFS were observed, although there was a trend toward a statistically significant difference in PFS between Classes 3 and 4 (HR 1.25 with 95% CI from 0.99 to 1.58 and *p*-value 0.064) (Table 2). Similar conclusions can be drawn from landmark analyses that removed patients who had events before 6 months, though the difference in the PFS between Classes 3 and 4 became statistically significant (HR 1.28 with 95% CI from 1.00 to 1.62 and *p*-value 0.048) (Figure S2 and Table S1). The C-index was 0.690 for the OS and 0.674 for the PFS.

## 4. Discussion

In the current study, we identified distinct classes of patients with ovarian cancer based on the different trajectories of CA125 within 3 and 6 months following chemotherapy treatment initiation. We investigated the association between the CA125 trajectory classes and the clinical outcomes and evaluated the prognostic value of the classification based on the association between the CA125 trajectory and the survival outcomes. Specifically, we found that the CA125 trajectories where values decreased after 3 or 6 months of treatment initiation or where the CA125 values were low at baseline and remained stable throughout treatment were associated with the lowest risk of death or progression. The patients with moderate CA125 values at baseline that decreased to normal values after treatment had a similar risk of death or progression. The patients with high CA125 values at baseline had a higher risk death or progression, with the highest risk observed in the patients whose CA125 did not normalize after treatment. The C-index values for the prognostic performance of the CA125 trajectory class for the OS were, respectively, 0.679 and 0.690, based on the CA125 values within 3 and 6 months of treatment. This was higher than the C-index of 0.639, which could be obtained if using only the baseline CA125 values.

Latent-class modeling has been used in studies for other type of cancers. For example, Li et al. [13] used the same approach that we used in this paper to identify subgroups of patients with distinct longitudinal CEA, CA19-9, and CA125 trajectories from a retrospective, longitudinal cohort of patients with colorectal cancer who underwent curative resection and investigated the overall survival and the recurrence-free survival of the patients in these groups. Zhang et al. [14] applied a growth mixture modeling (GMM) approach to identify classes of patients based on the longitudinal quality-of-life data collected from chemotherapy–refractory metastatic colorectal cancer (CRC) patients enrolled in the CCTG CO.20 clinical trial and evaluated for the differences between the classes with regard to their clinico-epidemiologic characteristics and their overall survival. To our knowledge, our study is the first to apply this methodology to patients with advanced ovarian cancer. We identified distinct classes of patients based on their longitudinal CA125 levels. Further investigation would apply a similar method to longitudinal outcomes of other biomarkers or patients’ quality of life.

Our study has both strengths and limitations. The data from our study are from a single clinical trial protocol and considered to be of high quality. However, this strength may limit the generalizability of the results to the general population, especially for patients who were treated with other chemotherapy regimens. The validation of our findings in other independent cohorts is needed for future research. Another limitation is that this study was based on data from a phase III trial completed in 2010. Although chemotherapy with a combination of carboplatin and paclitaxel is still used most often as the first-line treatment for advanced ovarian cancer, PARP-inhibitors are now available as a maintenance treatment. It would be of interest to verify the prognostic values of the CA125 trajectory classes using the data from trials with newly approved drugs, such as PARP-inhibitors, in a similar way to a study conducted recently, which assessed the KELIM’s prognostic and predictive values in PARP-inhibitor trials [7]. Another limitation is that the BRCA status could not be included in this analysis because the data on BRCA1 protein expression was only available for 251 of the patients enrolled in this study [15]. Based on our results, it would be of interest to measure the Ca-125 trajectory, as outlined in this our study, in patient populations receiving PARP-inhibitor maintenance therapy, stratified by their BRCA status.

Our findings suggest that dynamic measurements of CA125 taken during and at the end of chemotherapy treatment for patients with advanced ovarian cancer can identify the patients with a high risk for disease progression and death and, therefore, be used as an intuitive visual tool to monitor a patient’s prognosis. Similar methodology could be applied to develop trajectory classes based on other longitudinal measurements of biomarkers and patient-reported outcomes in other clinical settings.

## 5. Conclusions

The longitudinal trajectories of CA125 identified in this paper may provide more direct information for the prognoses of patients with newly diagnosed, advanced ovarian cancer undergoing first-line chemotherapy treatment after the completion of primary surgery or before interval debulking, and they may be a potential indicator of treatment efficacy. Longitudinal measurements of CA125 taken during and at the end of chemotherapy treatment for patients with advanced ovarian cancer can identify the patients at a high risk of disease progression and death and provide an intuitive visual tool for monitoring and informing their prognoses.

## Figures and Tables

**Table 1:** Baseline characteristics by 3-month CA125 trajectory class.

Variable	Class 1	Class 2	Class 3	Class 4	*p* Value
	(*n* = 236)	(*n* = 175)	(*n* = 226)	(*n* = 175)	
Age, median (range), years	58.9 [30.9, 78.3]	57.4 [30.2, 75.7]	57.6 [28.1, 75.6]	55.5 [30.6, 76.0]	0.018
ECOG performance status, No. (%)					<0.001
0	55 (23.3)	54 (30.9)	59 (26.1)	94 (53.7)	
1	181 (76.7)	121 (69.1)	167 (73.9)	81 (46.3)	
Residual disease, No. (%)					<0.001
None/micro	88 (37.3)	40 (22.9)	14 (6.2)	12 (6.9)	
Macro < 1 cm	30 (12.7)	31 (17.7)	77 (34.1)	43 (24.6)	
Macro ≥ 1 cm	99 (42.0)	82 (46.9)	66 (29.2)	36 (20.6)	
No debulking	13 (5.5)	21 (12.0)	67 (29.7)	82 (46.9)	
Unknown	6 (2.5)	1 (0.6)	2 (0.9)	2 (1.1)	
FIGO stage, No. (%)					<0.001
IIA	0 (0.0)	0 (0.0)	0 (0.0)	1 (0.6)	
IIB	2 (0.9)	1 (0.6)	4 (1.8)	14 (8.0)	
IIC	3 (1.3)	0 (0.0)	16 (7.1)	29 (16.6)	
IIIA	3 (1.3)	2 (1.1)	6 (2.7)	15 (8.6)	
IIIB	7 (3.0)	8 (4.6)	31 (13.7)	29 (16.6)	
IIIC	120 (50.9)	102 (58.3)	135 (59.7)	80 (45.7)	
IV	101 (42.8)	62 (35.4)	34 (15.0)	7 (4.0)	
Histology					<0.001
Serous adenocarcinoma	150 (36.4)	125 (71.4)	169 (74.8)	97 (55.4)	
Others	86 (63.6)	50 (28.6)	57 (25.2)	78 (44.6)	

While the patients in Class 4 were the youngest (median 55.5 years with a range from 30.6 to 76.0 years). Most of the patients in Class 1 and a minority of those in Class 4 had an ECOG performance status of 1 (76.7% and 46.3%, respectively); macro residual disease, which was 1 cm or larger (42.0% and 20.6%, respectively); and stage-IV disease (42.8% and 4%, respectively). Most of the patients in Class 3 and least in Class 1 had serous adenocarcinoma (74.8% and 36.4%, respectively).

**Table 2:** The multivariate Cox proportional hazard regression analysis for the association between the CA125 trajectory class and clinical outcomes.

	OS		PFS	
	HR (95%CI)	*p* Value	HR (95%CI)	*p* Value
3-month CA125 trajectory class				
Class 1	3.13 (2.37, 4.13)	<0.001	2.91 (2.26, 3.76)	<0.001
Class 2	1.86 (1.40, 2.46)	<0.001	1.88 (1.46, 2.44)	<0.001
Class 3	1.07 (0.82, 1.41)	0.614	1.13 (0.89, 1.43)	0.325
Class 4	Reference		Reference	
6-month CA125 trajectory class				
Class 1	4.83 (3.56, 6.54)	<0.001	5.15 (3.87, 6.87)	<0.001
Class 2	1.98 (1.52, 2.60)	<0.001	2.01 (1.57, 2.57)	<0.001
Class 3	1.18 (0.91, 1.54)	0.219	1.25 (0.99, 1.58)	0.064
Class 4	Reference		Reference	

**Table 3:** Baseline characteristics by 6-month CA125 trajectory groups.

Variable	Class 1	Class 2	Class 3	Class 4	*p* Value
	(*n* = 126)	(*n* = 256)	(*n* = 246)	(*n* = 184)	
Age (years), median (range)	58.4 [30.2, 78.3]	58.9 [35.5, 75.9]	57.1 [28.1, 75.3]	55.6 [30.6, 76.0]	0.003
ECOG performance status, No. (%)					<0.001
0	24 (19.1)	77 (30.1)	66 (26.8)	95 (51.6)	
1	102 (80.9)	179 (69.9)	180 (73.2)	89 (48.4)	
Residual disease, No. (%)					<0.001
None/micro	8 (6.3)	21 (8.2)	66 (26.8)	88 (47.8)	
Macro < 1 cm	20 (15.9)	37 (14.4)	80 (32.5)	44 (23.9)	
Macro ≥ 1 cm	53 (42.1)	111 (43.4)	80 (32.5)	39 (21.2)	
No debulking	39 (30.9)	85 (33.2)	18 (7.3)	12 (6.5)	
Unknown	6 (4.8)	2 (0.8)	2 (0.8)	1 (0.6)	
FIGO stage, No. (%)					<0.001
IIA	0	0	0	1 (0.5)	
IIB	2 (1.6)	1 (0.39)	4 (1.6)	14 (7.6)	
IIC	2 (1.6)	1 (0.39)	12 (4.9)	33 (17.9)	
IIIA	1 (0.8)	4 (1.56)	6 (2.4)	15 (8.2)	
IIIB	1 (0.8)	14 (5.47)	30 (12.2)	30 (16.3)	
IIIC	60 (47.6)	142 (55.47)	153 (62.2)	82 (44.6)	
IV	60 (47.6)	94 (36.72)	41 (16.7)	9 (4.9)	
Histology, No. (%)					<0.001
Serous adenocarcinoma	75 (59.5)	177 (69.1)	187 (76.0)	102 (55.4)	
Others	51 (40.5)	79 (30.9)	59 (24.0)	82 (44.6)	

## Data Availability

The raw data supporting the conclusions of this article will be made available by the authors on request.

## Supplementary Materials

The following supporting information can be downloaded at https://www.mdpi.com/article/10.3390/curroncol32070390/s1. Figure S1: Kaplan–Meier curves by 3-month CA125 trajectory class for OS (left) and PFS (right) from landmark analysis. Table S1: A landmark analysis by a Cox proportional hazard regression analysis for the prediction of clinical outcomes by the CA125 trajectory class. Figure S2: Kaplan–Meier curves by 6-month CA125 trajectory class for OS (left) and PFS (right) from landmark analysis.
