# Supplementary material for: Trajectories of Cancer Antigen 125 (CA125) Within 3 and 6 Months After the Initiation of Chemotherapy Treatment for Advanced Ovarian Cancer and Clinical Outcomes: A Secondary Analysis of Data from a Phase III Clinical Trial
Source: Curr Oncol. 2025 Jul 7;32(7):390. doi: 10.3390/curroncol32070390 (PMC12293772; doi:10.3390/curroncol32070390)
Supplement: Supplementary file 1 [file curroncol-32-00390-s001.zip › curroncol-3676067-supplementary.pdf]

Supplemental Figures and Tables for "Trajectories of Cancer Antigen 125 (CA125) During 3 and 6 Months After Initiation of Chemotherapy Treatment for Advanced Ovarian Cancer and Clinical Outcomes: A Secondary Analysis of Data from a Phase III Clinical Trial"

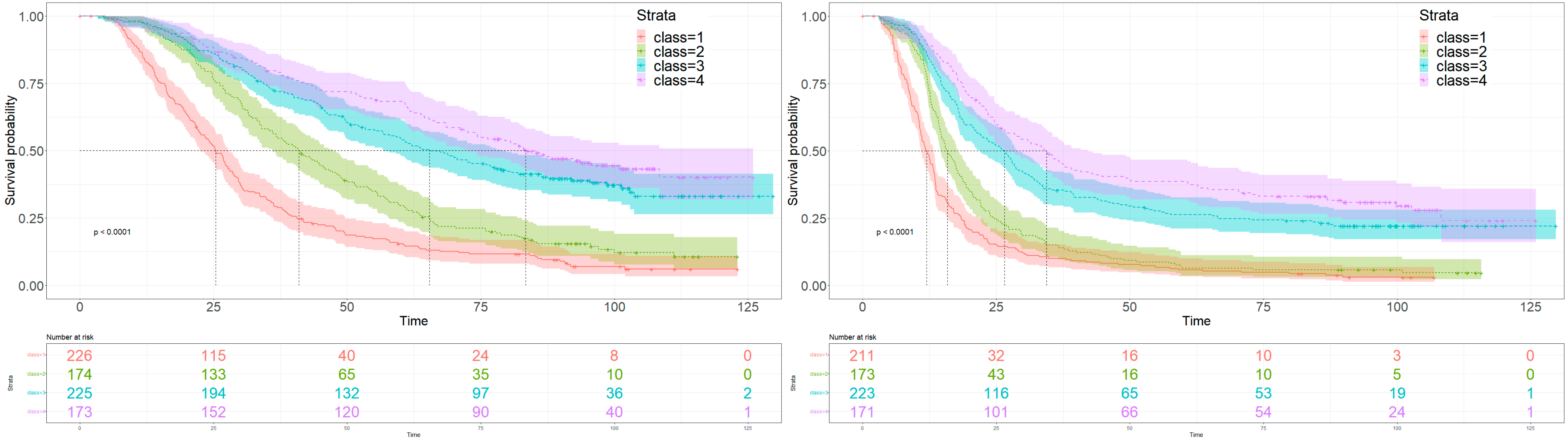

Figure S1. Kaplan-Meier Curves by 3-month CA125 trajectory class for OS (left) and PFS (right) from landmark analysis

Table S1. Landmark analysis by Cox proportional hazard regression analysis for the prediction of clinical outcomes by CA125 trajectory class.

	OS		PFS	
	HR (95%CI)	*P* value	HR (95%CI)	*P* value
**3-month CA125 trajectory class**				
Class 1	3.22 (2.45, 4.25)	<0.001	2.80 (2.16, 3.62)	<0.001
Class 2	1.92 (1.45, 2.54)	<0.001	1.90 (1.47, 2.46)	<0.001
Class 3	1.11 (1.45, 2.54)	0.455	1.15 (0.90, 1.47)	0.253
Class 4	Reference		Reference	
**6-month CA125 trajectory class**				
Class 1	4.93 (3.63, 6.69)	<0.001	3.98 (2.88, 5.49)	<0.001
Class 2	1.98 (1.51, 2.60)	<0.001	1.98 (1.54, 2.55)	<0.001
Class 3	0.19 (0.91, 1.55)	0.198	1.28 (1.00, 1.62)	0.048
Class 4	Reference		Reference	

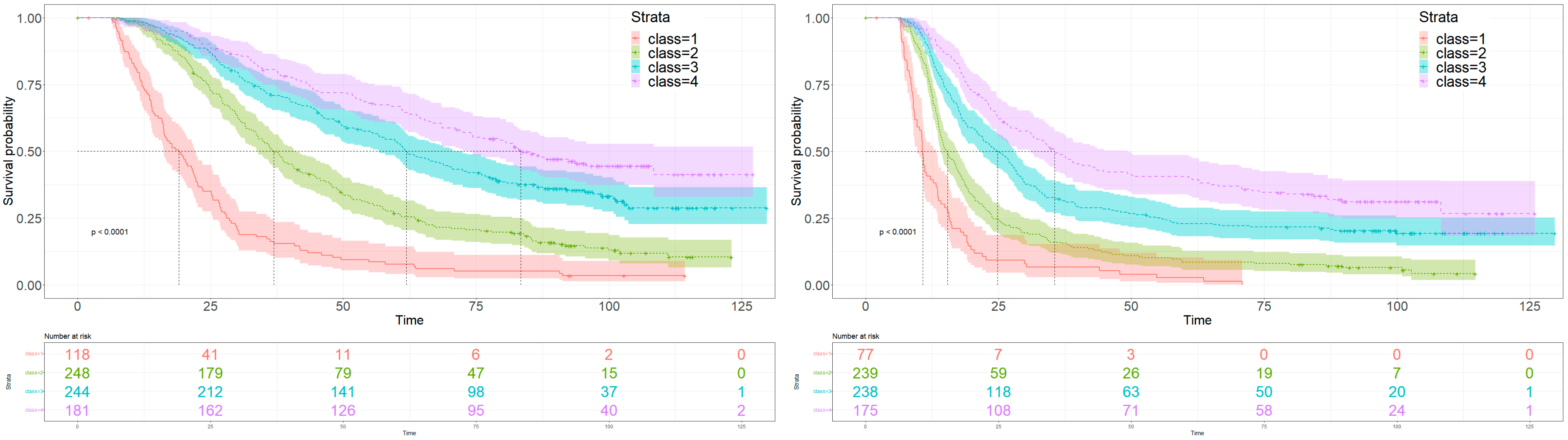

Figure S2. Kaplan-Meier Curves by 6-month CA125 trajectory class for OS (left) and PFS (right) from landmark analysis
